# Psychosocial interventions for children and adolescents across the cancer care continuum: A systematic review and meta-analysis

**DOI:** 10.1007/s00520-026-11176-x

**Published:** 2026-09-21

**Authors:** Catherine Paterson, Maria C. McCarthy, Ursula M. Sansom-Daly, Claire E. Wakefield, Murray R. Turner, Juliana Christina, Cara Roberts, Cameron Moss, Carla Thamm, Mary-Ann Carmichael, Safora Johansen, Morgan Atkinson, Michael Osborn, Ben Singh, Imogen Ramsey

**Affiliations:** 1 Flinders University, College of Nursing and Health Sciences, Caring Futures Institute, Adelaide, Australia; 2 Central Adelaide Local Health Network, Adelaide, Australia; 3 Murdoch Children’s Research Institute, Melbourne, Australia; 4 Children’s Cancer Centre, Royal Children’s Hospital, Melbourne, Australia; 5 Department of Paediatrics, University of Melbourne, Melbourne, Australia; 6 Behavioural Sciences Unit, School of Clinical Medicine, Discipline of Paediatrics and Child Health, Randwick Clinical Campus, UNSW Medicine and Health, UNSW, Randwick, NSW Australia; 7 Kids Cancer Centre, Sydney Children’s Hospital, Randwick, Australia; 8 Sydney Youth Cancer Service, Nelune Comprehensive Cancer Centre, Prince of Wales Hospital, Randwick, Australia; 9 Division of Quality of Life and Pediatric Palliative Care, Department of Pediatrics, Stanford University and Stanford Medicine Children’s Health, Palo Alto, CA USA; 10 University of Canberra, Canberra, ACT Australia; 11 Canberra Health Services, Canberra, Australia; 12 Oslo Metropolitan University, Health Faculty, Oslo, Norway; 13 Oslo University Hospital, Department of Cancer Treatment, Oslo, Norway; 14 Singapore Institute of Technology, Health and Social Science Cluster, Punggol, Singapore; 15 Women’s and Children’s Hospital, North Adelaide, Australia; 16 University of Adelaide, Adelaide, Australia; 17 Alliance for Research in Exercise Nutrition and Activity (ARENA), University of South Australia, Adelaide, South Australia Australia

**Keywords:** Psychosocial interventions, Cancer, Care continuum, Systematic review, Meta-analysis, Children, Adolescents

## Abstract

**Purpose:**

To evaluate the impact of psychosocial interventions on health outcomes among children and adolescents (0–19 years) living with cancer across the care continuum, specifically on (1) neurocognitive, (2) psychological, and (3) health-related quality of life (HRQOL) outcomes.

**Methods:**

Five electronic databases (APA PsycINFO, CINAHL, MEDLINE, Scopus and the Cochrane Central Register of Controlled Trials [CENTRAL]) were searched with no date restrictions, supplemented by backward citation chaining of the reference lists of included studies and relevant systematic reviews. All identified citations were imported into Covidence systematic review software for the removal of duplicate records and the study selection process by two reviewers. Methodological quality and meta-analyses were performed.

**Results:**

Two thousand nine hundred sixty-one articles were identified, and 34 studies were included. The studies were conducted in 17 countries and representative of 1988 participants. Studies investigating the effects of cognitive interventions demonstrated a significant positive effect on neurocognitive outcomes (SMD: 0.51 [95% CI: 0.32, 0.69]; *I*^2^ = 0%, *p* < 0.01). Play and creative therapies, animal assistance therapy, self-management education, and physical activity were associated with a significant overall improvement in psychological outcomes (SMD: 0.42 [95% CI: 0.17, 0.68]; *I*^2^ = 70.1%, *p* < 0.01). Animal assistance therapy, play and creative therapies, and physical activity interventions led to a significant improvement in quality-of-life outcomes (SMD: 0.80 [95% CI: 0.48, 1.11]; *I*^2^ = 87.1%, *p* < 0.01).

**Conclusions:**

Psychosocial interventions improved neurocognitive, psychological, and HRQOL outcomes in children and adolescents with cancer, with the most consistent benefits for neurocognition (working memory), anxiety, and pain; effects on depression, coping, self-worth, fatigue and global HRQOL were not significant. Future trials should identify optimal intervention timing, dosage and delivery format, and stratify outcomes by age, diagnosis, and treatment intensity.

**Implications for cancer survivors:**

These findings suggest the promise of interventions for improving neurocognitive, psychological, and HRQOL outcomes in children and adolescents affected by cancer, while also highlighting substantial evidence gaps, particularly for early targeted approaches in the cancer care continuum.

**Supplementary Information:**

The online version contains supplementary material available at https://doi.org/10.1007/s00520-026-11176-x.

## Introduction

The incidence of cancer in children has increased over the past 40 years by 13% [1], such that globally, approximately 400,000 children under the age of 19 years are diagnosed with cancer each year [2]. A recent systematic review [3] identified that children and adolescents continue to experience a range of unmet supportive care needs despite routine contact with healthcare professionals, underscoring the need for further targeted interventions. Children and adolescents will often receive invasive surgery, intensive chemotherapy, and radiotherapy regimens. Beyond the physical burden of treatment, a substantial proportion of children and adolescents with cancer experience clinically significant psychosocial morbidity. In a longitudinal paediatric cohort, 48% met criteria for an anxiety and/or depressive disorder at least once during the first year following diagnosis [4], and a meta-analysis of adolescent and young adult survivors estimated pooled prevalences of 29% for anxiety and 24% for depression [5]. Relative to matched controls and siblings, young people treated for cancer carry an elevated lifetime risk of depression (RR 1.57), anxiety (RR 1.29), and trauma- and stressor-related disorders [6]. Neurocognitive late effects are similarly common: global neurocognitive impairment has been reported in almost half (47%) of survivors treated with CNS-directed therapy, predominantly affecting attention, working memory, processing speed and executive function [7]. These difficulties are not benign. Left unaddressed, psychological distress is associated with poorer adherence to cancer treatment, reduced survival and impaired health-related quality of life (HRQoL). Together, these data establish both the scale of psychosocial need and the clinical imperative for timely, targeted intervention across the cancer care continuum [8].

The term psychosocial refers to the dynamic interplay between an individual’s internal psychological processes, such as cognition, emotion, and behaviour, and the social worlds in which they exist, including family relationships, peers, school, and healthcare systems [9]. The psychosocial consequences of cancer treatment have been shown to directly negatively impact HRQoL, self-esteem, sleep, school attendance, and problems in the development and maintenance of relationships with family and friends [10, 11]. A recent systematic review [12] identified that childhood and adolescent cancer survivors are at increased risk of social development delays and disruptions, due to physical and psychological changes which increase the risk of social isolation and loneliness. Peer relationships are a protective factor associated with improved social functioning over the cancer continuum [12], however for some young people, these relationships are also negatively affected [3].

Following a cancer diagnosis, many children and adolescents report that they want to be supported to make healthier food choices [13], regain physical and psychological wellbeing [14, 15], have good support structures for optimising supported self-management [15–19] and build independence and resilience strategies [20–22], which is particularly important for teenagers [17, 18, 20, 22] in coping with returning to normal life. Many young people have also reported to have a high need for timely access to counselling and psychological services, in tandem with individualised information and advice, and social relationships support over the cancer care continuum [23]. It is clear that introducing early and targeted interventions to prevent rather than react to negative impacts that cancer and treatments would be beneficial.

Psychosocial interventions are targeted interventions that address both the emotional and social aspects of one’s well-being. Types of psychosocial interventions in children and adolescents can include both structured and unstructured modalities, drawing from cognitive behaviour therapy, skills training (social and behavioural), social/support groups, play therapy, music therapy, art therapy, family therapy, psycho-education, and counselling [24, 25]. To the best of our knowledge [25, 26], there has not been a contemporary critical synthesis of all psychosocial interventions tested in children and adolescents < 19 years of age from diagnosis into survivorship [27] with a focus on (1) psychological, (2) neurocognitive, and (3) quality of life outcomes to provide a broad comprehensive understanding of the existing evidence landscape.

More recent systematic reviews and meta-analyses [24, 28] have been conducted in relation to psychosocial interventions among childhood cancer survivors but have been limited by (1) a very limited number of randomised controlled studies (RCTs), (2) inclusive of long-term survivors who are > 35 years old susceptible to recall bias for their experiences prior to the age of 19, (3) existing reviews did not include psychosocial interventions inclusive of the cancer care continuum from diagnosis into survivorship, an important omission in the development of seamless integrated psychosocial care over time, (4) a lack of insight into how psychosocial interventions may impact neurocognitive outcomes related to brain function and cognitive processes such as thinking, memory, and decision-making, and finally, (5) limited understanding about which identified factors informed the psychosocial development of interventions, to understand the theory-to-design map that should guide aged and developmentally targeted content and delivery tailored across the age span (0–19 years) [29].

Therefore, there is an important clinical need to build upon the evidence to understand the impact of psychosocial interventions among young people living with cancer from diagnosis into survivorship. To the best of the authors’ knowledge, to date, this is the most comprehensive review and meta-analysis of psychosocial interventions across the cancer care continuum for young people. This systematic review addressed the following clinically focused research question:

1. How effective are psychosocial interventions for children and adolescent (0–19 years) diagnosed with cancer compared to standard care on (1) neurocognitive, (2) psychological, and (3) health-related quality of life outcomes?

## Methods

This systematic review and meta-analysis were reported according to the Systematic Reviews and Meta-Analysis (PRISMA) 2020 statement [30] (see Supplementary Table 1 for completed PRISMA checklist). This review followed an a priori protocol developed before screening commenced. The protocol was not prospectively registered in a public database; it is available from the corresponding author on request.

### Inclusion and exclusion criteria

Randomised or quasi-randomised trials (with a comparator arm) investigating interventions, comprising all children who had been diagnosed with cancer (0–19 years of age), were included. If the studies had mixed samples of participants with other long-term conditions, the studies were included if the samples were > 50% cancer and reported outcomes on people with cancer to be included in analysis. Only studies reported in the English language were included. The Participants, Intervention, Comparator, and Outcome (PICO) framework [31] was used to develop the eligibility criteria as follows: participants: studies of children and adolescents (0–19 years) diagnosed with cancer, regardless of stage, treatment regimen, and stage in the cancer care continuum were included. Studies focused on parents or siblings were excluded. Intervention: studies of psychosocial interventions were included in prehabilitation (before cancer treatments) and rehabilitation (during and after treatment into survivorship) phases of the cancer care continuum. Interventions were defined as any form of uni- or multi-modal intervention designed to improve neurocognitive, psychological, and health-related quality of life outcomes. Psychosocial interventions for children with cancer are non-pharmacological strategies these interventions excluded medical, pharmacological, and lifestyle-focused treatments unless they are explicitly delivered within a psychological or behavioural framework. Pharmacological interventions (e.g., antidepressants, sedatives), sleep and fatigue interventions (unless integrated within a psychological and behavioural framework), nutritional or physical rehabilitation programs (unless integrated with psychosocial goals), and educational interventions (focused solely on academic performance without a psychosocial component) were excluded. Comparator: studies with comparators including usual care, waitlist controls, other interventions, and healthy children were included. Outcomes: studies reporting data on cognitive outcomes, assessed using neuropsychological tests, as well as patient- and/or proxy-reported outcomes such as health-related quality of life (HRQOL), symptoms, and psychosocial outcomes using patient-reported outcomes were included.

### Search strategy

A search strategy was designed by an expert librarian (MT), utilising the efficient search method for systematic reviews developed at Erasmus University Medical Centre [32]. The search used a combination of keywords and subject headings (see Supplementary Table 2 for the full record of database searches).

The APA PsycINFO, CINAHL, Medline, and Scopus databases, and the Cochrane Central Register of Controlled Trials (CENTRAL) were searched in May 2023 and updated in November 2025 to identify relevant studies with no publication age limiters. A range of keywords and subject headings were utilised to capture the concepts of psychosocial interventions, cancer, and children and adolescents. Search filters adapted from the CADTH Search filters database (https://searchfilters.cadth.ca/) were applied to retrieve trials. To capture all relevant studies, no date restrictions were placed on the searches. Relevant systematic reviews and the reference lists of the included studies were scrutinized for potentially additionally relevant studies for screening. Search results were managed with Endnote software.

### Study selection

The screening process was managed using Covidence Systematic Review Software. Two review authors independently screened all titles and abstracts of identified records according to the inclusion criteria. A third reviewer resolved all conflicts. The full texts of all potentially eligible records were retrieved and screened independently by two authors. Any conflicts were resolved by a third reviewer via discussion.

### Data extraction

Study characteristics were extracted by several authors and cross-checked for accuracy. Data were extracted and included the following: author and year; study design, setting, and country; intervention characteristics; sample size; participant age, gender, and cancer type; outcome measures; and statistics for effect size calculations (i.e., means and standard deviations, *p*-values, *F* statistics).

### Critical appraisal

Risk of bias of the included sources was assessed by one author using the Joanna Briggs Institute critical appraisal checklists, appropriate for each study design [33]. Assessments were checked by a second reviewer, and any disagreements were resolved by discussion. Each assessment considered the randomisation, allocation concealment, blinding, completeness of outcome data, selective reporting, and any other potential bias. Studies were categorised as being at low, moderate, or high overall risk of bias according to the proportion of applicable JBI criteria met (≥ 70%, 50–69%, and < 50%, respectively).

### Statistical analysis

Meta-analyses were performed for outcomes reported in more than one RCT. For studies with multiple publications, only the most recent or complete data for each outcome was used. Outcomes of interest were analysed as continuous variables, and data were pooled using either (i) post-intervention means and standard deviations (SDs) for the intervention and control groups. Standardised mean differences (SMDs) were used as the effect measure for meta-analyses, to allow comparison of data from different scales. If means and SDs were not reported in a study, authors were contacted or means and SD were calculated based on available data, using recommended formulas (e.g., using sample size, median and range), or the study was not used in the pooled analysis. A random effects model was used to account for heterogeneity among studies. Both the classical Cochran’s Q measure of heterogeneity and the *I*^2^ statistic were used to quantify the percentage of variation across studies due to heterogeneity rather than chance [34–36]. Publication bias was not assessed using funnel plots or formal tests for small-study effects because each meta-analysis included fewer than 10 studies. In this context, funnel plot asymmetry and statistical tests such as Egger’s test are underpowered and may provide misleading results, particularly given the clinical and methodological heterogeneity across studies. Standardised classifications for the magnitude of effect sizes were used as follows: 0.20 representing a small effect; 0.50 representing a medium effect; and greater than 0.80 representing a large effect [37]. A *p*-value of < 0.05 was considered statistically significant. All meta-analyses were performed using Stata (Version 18).

We considered whether developmental age could be explored through subgroup analysis or meta-regression. This was not undertaken because most trials reported age only as aggregate study-level data, and many recruited across broad, overlapping age ranges spanning multiple developmental stages. Assigning whole studies to single age strata would therefore risk misclassification and ecological bias. In addition, the small number of independent studies per outcome, combined with variation in intervention type, outcome measure, diagnosis, and treatment phase, meant that age-based meta-regression would be underpowered and potentially misleading. Age was therefore examined narratively, and pooled effects were interpreted in the context of this clinical heterogeneity. We also considered whether heterogeneity in psychological and HRQOL outcomes could be explored using subgroup, meta-regression or sensitivity analyses. These analyses were not undertaken because the number of independent studies per outcome was small, several pooled estimates included multiple outcomes from the same studies, and potential subgroups by intervention type, cancer type, or developmental age were sparse and overlapping. Intervention modality, diagnosis, age range, treatment phase, comparator, delivery format, and outcome measure were also highly interrelated, making it difficult to isolate any single source of heterogeneity. Additional post hoc analyses were therefore considered underpowered and potentially misleading. Heterogeneity was instead examined narratively and incorporated into the interpretation of pooled effects.

## Results

**Fig. 1:**
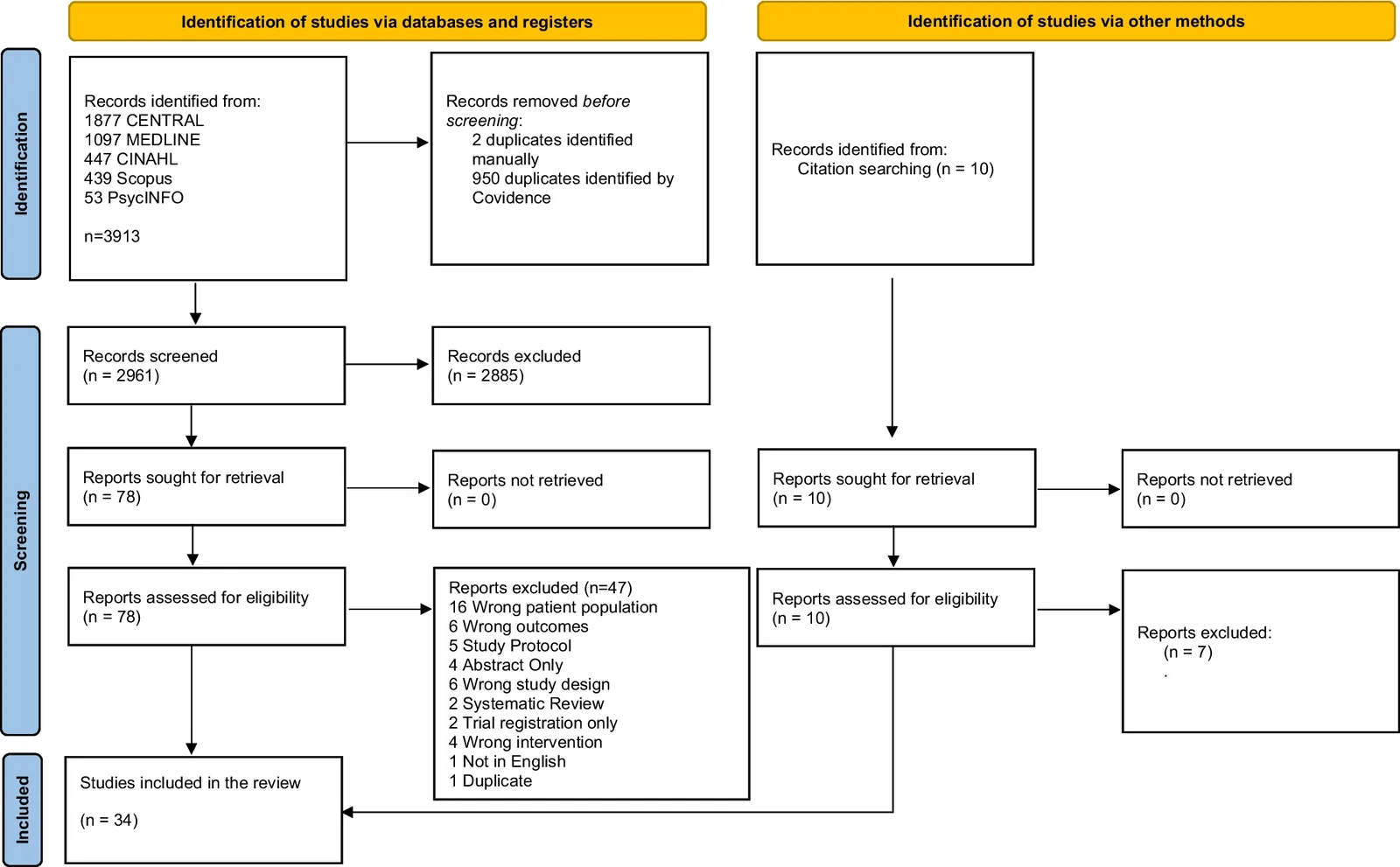
PRISMA flow diagram

Of the 2961 articles screened, a total of 78 articles were reviewed in full, Fig. 1 for PRISMA diagram. A total of 34 studies were included. The studies were conducted in a range of countries which included the folowing: Australia (1), France (1), Germany (2), China (1), Iraq (1), Iran (1), Indonesia (1), Netherlands (1), Singapore (1), Saudi Arabia (1), Spain (2), Sweden (1), Switzerland (1), Taiwan (1), Turkey (6), and the United States of America (13). The sample sizes ranged from 13 to 178 with a total of 1988 participants represented, see Table 1. Largely, the samples were clinically heterogeneous cancer samples [38–58], leukaemia [59–61], and brain cancer [62, 63], and two studies did not report on cancer type [64, 65]. There were no early intervention (prehabilitation) psychosocial clinical trials conducted prior to commencing treatment. All the trials included a variety of interventions, namely, art therapy [45, 64], jewellery making [60], cognitive rehabilitation [38, 39, 41, 43, 46, 63], child life [66], physical and psychosocial training [40, 44, 54, 55, 61, 65], symptom management [42], aqua therapy [59], web-based and game physical activity [47, 62], immersive virtual reality [67, 68], self-management education [48, 56–58], animal-assisted therapy [49], play therapy [50], complementary therapies [51], massage therapy [52], and music therapy [53, 69]. The intervention duration lasted from one single session to 24 weeks.

**Table 1:** Study overview

Study details		Intervention characteristics					Methods		Participants	
Author, year	Country	Theoretical basis	Provider	Setting	Description	Frequency and duration	Comparator	Outcome measures	N	Age range
Abdulah et al. 2018	Iraq	None	Artist	Hospital	Painting and handcrafting group art therapy	20 sessions over 4 weeks	Usual care	KIDSCREEN-10	60	7–13
Akel et al. 2019	Turkey	None	Occupational therapist	Hospital	Cognitive rehabilitation (cognitive skills, spatial perception, praxis, attention, thinking operations)	15 sessions, duration not reported	Routine OT program	WeeFIM	40	6–12
Benzing et al. 2020	Switzerland	None	Psychologist or trained postgraduate student	Home-based	Cogmed working memory training program, or exergaming (Shape UP)	3 sessions weekly over 8 weeks	Waitlist control	WMTB-C: Block Recall Test, D-FEKS: Colour Word Interference Test	69	7–16
Braam et al. 2018	Netherlands	None	Physical therapists and psychologist	Physiotherapy practice with additional home-based program after week 6	Physical exercise sessions and psychosocial training (psychoeducation and CBT)	2 physical exercise sessions weekly and 1 psychosocial training session weekly for 12 weeks	Usual care	PedsQL Multidimensional Fatigue Scale and Generic Core Scales, SPP, YSR, CDI	68	8–18
Butler et al. 2008	USA	None	Graduate clinical psychology students, equivalent healthcare professional, or trained researchers	Hospital	Attention Process Training cognitive rehabilitation program	22 weekly sessions over 4–5 months	Waitlist control	CPT-II, CPRS:LV-R, WRAT-3, PIAT-R, WJ-R, WISC-III, RAVLT, CFSEI	161	6–17
Cheng et al. 2021	Singapore	Social Cognitive Theory and Symptom Cluster Concept	Trained research assistant	Home-based	Multimodal symptom management program (psychoeducation and coaching in relaxation, distraction, and lifestyle strategies)	Initial visit then weekly phone calls for the duration of chemotherapy	Usual care	MSAS 10–18, CSAS	50	10–18
Chubak et al. 2024	USA	None	Therapy dog handlers	Hospital	Animal therapy visits from a therapy dog	Weekly for up to f4 weeks	Usual care	PedsQL, PANAS, STAI,	26	5–17
Conklin et al. 2013	USA	None	Not reported	Home-based	Cogmed working memory training program	25 training sessions over 9 weeks with weekly telephone coaching	Waitlist control	WISC-IV, CPRS-3, BRIEF, CPT-II, WJ-III	62	8–16
Elnaggar et al. 2021	Saudi Arabia	None	Paediatric physical therapist	Physical rehabilitation center	Lower-body plyometric exercise program	3 sessions weekly for 12 weeks	Usual care	PedsQL Generic Core Scales	30	8–18
Fiuza-Luces et al. 2017	Spain	None	Fitness Instructors	Hospital	Supervised exercise program (resistance and aerobic)	3 sessions weekly for (19 ± 2 weeks)	Usual care	PedsQL Generic Core Scales	59	4–17
Günay et al. 2022	Turkey	None	Researcher	Hospital	Group-based jewellery-making activity using beads	2 sessions weekly for 4 weeks	Usual care	STAI	62	7–18
Gürcan et al. 2021	Turkey	None	Research assistant	Hospital	Mandala drawing activity	2 sessions within a week	Usual care	HADS	60	12–17
Hardy et al. 2013	USA	None	Self-directed with weekly check-ins from a treatment “coach”	Home-based	CogmedRM working memory training program	3–5 sessions weekly for 5–8 weeks	Active control (nonadaptive cognitive training program)	WRAML2, CPRS-3	20	8–16
Howell et al. 2018	USA	None	Self-directed	Home-based	Physical activity intervention involving education, activity monitoring, and an interactive website	Self-paced engagement over 24 weeks	Active control (education and activity monitor)	PedsQL,	78	11–15
Kudubes et al. 2019	Turkey	None	Not reported	Hospital	Fatigue-related education program for parents and children	8 sessions, duration not specified	Waitlist control	Symptom Assessment Scale for Patients, Scale for the Assessment of Fatigue in Pediatric Oncology Patients aged 7–12 (child and parent forms), Scale for the Quality of Life in Pediatric Oncology Patients Aged 7–12 (child and parent forms)	80	7–12
Li et al. 2023	China	Children’s stress and coping	Researchers	Hospital	Child Life (Theories of children’s stress and coping, cognitive development, family support, and play-based learning)	30–30 min/session 2 × week for 2 months	Usual care	Ped-PROMIS	96	8–14
McCullough et al. 2018	USA	None	Registered therapy dog-handler team	Hospital	Animal therapy visits from a therapy dog	1 session weekly for 16 weeks	Usual care	PedsQL, STAI	106	3–17
Mehling et al. 2012	USA	None	Massage practitioners	Hospital	Combined Swedish and acupressure massage	3 massages weekly for an average stay of 40 days	Usual care	BASES, PedsQL	25	5–18
Mohammadi et al. 2021	Iran	None	Occupational therapist	Hospital	Play therapy involving play-based occupational therapy and free play	8 sessions over 2 weeks	Usual care	TRSC, CPAS-P, FAS, FPS	25	7–12
Phipps et al. 2010	USA	None	Massage therapists and research assistants for the humour component	Hospital	Massage, humour materials, and education about the benefits of both	3 massages weekly for 3 weeks	Usual care	BASES, PANAS-C	178	6–18
Post-White et al. 2009	USA	None	Massage therapists	Hospital	Massage for children and parents	1 session weekly for 4 weeks	Quiet time	STAI, FACES, Child Fatigue Scale	25	1–18
Reitze et al.2024	Germany	None	Nursing staff and physicians	Paediatric oncology outpatient clinic	Virtual Reality	Not reported	Usual care	SSKJ3-8R, PedsQL, NRS, FPS-r, mYPAS-SF, BAADS	38	6–18
Robb et al. 2017	USA	None	Music therapists	Hospital, with home-based materials also provided	Active music engagement with parent education/coaching	3 sessions over 1 week	Active control (audiobook)	POMS-SF (parent emotional distress)	16	3–8
Sabel et al. 2017	Sweden	None	Self-directed, with weekly coaching provided by a research nurse	Home-based	Active gaming using Nintendo Wii and a Wii Fit balance board	5 sessions weekly for 10–12 weeks	Waitlist control	AMPS, CPT-II, D-KEFS, RAVLT, WISC-IV	13	7–17
SanJuan et al. 2008	Spain	None	Exercise instructor	Hospital	Supervised exercise training program	3 sessions weekly for 8 weeks	Healthy controls matched for age and gender	CHIP-CE	16	8–16
Sahin et al. 2025	Turkey	None	Parents	Hospital	Ocean Wave and LED Starry Sky Projector	Nightly for 1 week	Usual care	FIS, PedsQL	60	8–18
Senn-Malashonak et al. 2019	Germany	None	Not reported	Hospital	Supervised exercise training program (resistance, endurance, and flexibility)	5 weekly sessions	Mental and relaxation training	KINDL	70	5–18
Smith et al. 2022	USA	None	Physical therapist	Hospital	Supervised exercise program	1–3 sessions weekly for 6 weeks	Usual care	PROMIS Pediatric-49, WeFIM	40	4–18
Speyer et al. 2010	France	None	Physical activity teacher	Hospital	Individualised physical activity, with options including ball sports, circus arts, throwing/shooting games, racket sports, video games, fighting activities, body building and self-expression through movement	3 sessions weekly for duration of stay (average 14 days)	Crossover	CHQ	30	5–18
Sriasih et al. 2019	Indonesia	None	Nurse	Cancer lodges	Combined music and sleep hygiene education	4 days	Usual care	Barthel Index	58	7–18
Tennant et al. 2020	Australia	None	Trained research assistant	Hospital	Immerse Virtual Reality (VR)	10 min virtual simulation	Non-immersive iPad	VASs, SpO_2_, PedsQL, SCAS, VAS – Enjoyment thermometer, Adapted version of the *Total Immersion* subscale of the CCSQ	90	7–19
Tyc et al. 1997	USA	None	Psychologist	Hospital	CBT (modelling, breathing exercises, imagery, behavioural rehearsal, positive incentive)	Single session	Usual care	STAI, distress	55	6–18
Wu et al. 2019	Taiwan	Self-efficacy theory	Research assistant	Hospital	Self-management educational intervention	6 sessions within 1 week	Usual care	HBSE, HPL	64	8–18
Wu et al. 2014	Taiwan	Lazarus’ transactional theory	Research assistant	Hospital	Psychoeducational symptom-management intervention	1 week	Not described	PSS	58	Not reported

*AMPS* assessment of motor and process skills, *BAADS* Behavioral Approach-Avoidance and Distress Scale, *BASES* Behavior and Symptom Evaluation System, *BRIEF* Behavior Rating Inventory of Executive Function, *CDI* Children’s Depression Inventory, *CFSEI* Culture-Free Self-Esteem Inventory, *CHIP-CE* Child Health and Illness Profile–Child Edition, *CHQ* Child Health Questionnaire, *CPRS-3* Conners’ Parent Rating Scale, 3rd edition, *CPRS:LV-R* Conners’ Parent Rating Scale: Long Version–Revised, *CPT-II* Conners’ Continuous Performance Test–II, *CSSQ* Child Simulation Sickness Questionnaire, *C-Ped-PROMIS* The Chinese version of Paediatric Patient-Reported Outcomes Measurement Information System, *D-KEFS* Delis-Kaplan Executive Function System, *FAS* Faces Anxiety Scale, *FPS* Wong-Baker Faces Pain Rating Scale, *FIS* Fatigue Intensity Scale, *HADS* Hospital Anxiety and Depression Scale, *HBSE* Health Behaviour Self-Efficacy Scale, *HPL* Health Promotion Lifestyle Scale, *KINDL *Questionnaire for Measuring Health-Related Quality of Life in Children and Adolescents, *MSAS 10–18* Memorial Symptom Assessment Scale, *mYPAS* The modified Yale Preoperative Anxiety Scale, *NRS *Numeric Rating Scale, *PANAS-C* Positive and Negative Affect Schedule for Children, *PedsQL* Pediatric Quality of Life Inventory, *PIAT-R* Peabody Individual Achievement Test–Revised, *POMS *Profile of Mood States, *POMS-SF *Profile of Mood States–Short Form, *PROMIS *Patient-Reported Outcomes Measurement Information System, *PSS* Perceived Symptom Severity Scale, *RAVLT* Rey Auditory Verbal Learning Test, *SCAS* Spence Children’s Anxiety Scale, *SDSC* Sleep Disturbance Scale for Children, *SPP* Children’s Self-Perception Profile, *STAI* State-Trait Anxiety Inventory, *SSKJ 3–8 R* Stress und Stressbewältigug im Kindes- und Jugendalter-Revision, *TRSC* Therapy-Related Symptom Checklist, *VASs* Visual Ananlog Scales, *WISC-III* Wechsler Intelligence Scale for Children, 3rd edition, *WISC-IV* Wechsler Intelligence Scale for Children, 4th edition, *WJ-III* Woodcock-Johnson Tests of Achievement, 3rd edition, *WJ-R* Woodcock-Johnson Tests of Achievement, Revised, *WRAT-3* Wide Range Achievement Test, 3rd edition, *WRAML2* Wide Range Assessment of Memory and Learning, 2nd edition, *WMTB-C* Working Memory Test Battery for Children: Block Recall Test, *WeeFIM* Functional Independence Measure for Children, *YSR* Youth Self-Report

Of the studies reviewed [38–40, 43–47, 49–52, 59, 64], most did not report the use of a theoretical framework to guide intervention development. Only four studies explicitly referenced such models: one employed Social Cognitive Theory alongside Symptom Cluster Concepts [42], another utilized Self-efficacy Theory [57], and a third applied Lazarus’ Transactional Theory [58, 66]. Although too few studies were theoretically informed to support definitive conclusions, the models identified map onto distinct intervention purposes and developmental stages. Social Cognitive and Self-efficacy theories underpinned skills-based, autonomy-supportive interventions such as self-management education; these models are best suited to older children and adolescents, who have the cognitive capacity for goal-setting, self-monitoring and behaviour change, and are therefore most applicable to the survivorship and rehabilitation phases where self-management of health behaviours and re-establishing independence are central. Lazarus’ transactional model of stress and coping underpinned appraisal- and coping-focused interventions and is most relevant where the target is acute, treatment-related distress, making it applicable across the age range but particularly during active treatment and around discrete procedures. Symptom Cluster Concepts, applied alongside Social Cognitive Theory in one study [42], suit symptom-management interventions during the treatment phase. The predominance of atheoretical, “one-size-fits-all” designs across the remaining studies, with no developmental tailoring, co-design or theory-driven intervention development remained absent.

There was also a notable lack of acknowledgement of age-appropriate considerations for the intervention design and delivery content [38–40, 43–47, 49–52, 59, 64], and these observations were underscored by a very limited reporting of co-design methodologies across all the intervention studies with children (0–19 years old), family members, and healthcare professionals [38–40, 42–47, 49–52, 59, 64]. There was the exception of one study [48] which elicited feedback from healthcare professionals on educational content, but not from children or their families. A one-size-fits-all approach to psychosocial intervention delivery was observed for children across the following age groups: 1–18 years [52], 3–8 years [53], 3–17 years [49], 4–17 years [44], 5–17 [70], 5–18 years [54, 55, 71], 6–12 years [38], 8–14 years [66], 6–18 years [51, 63, 68], 7–12 years [48, 50], 7–13 years [64], 7–16 years [39, 41], 7–17 years [62], 7–18 years [56, 60], 8–16 years [43, 46], 8–18 years [69], 10–18 years [42], 11–15 years [47], 12–17 years [45], 8–16 years [61], 8–18 years [40, 57, 59], 7–19 years [67]. Given these broad and overlapping age ranges, and the absence of developmental-age-specific effect estimates, studies could not be allocated reliably to age strata for quantitative subgroup analysis.

The results of the methodological quality assessment are presented in Table 2. Overall, methodological quality was moderate. Of the 34 included studies, 15 (44%) were judged to be at low risk of bias, 18 (53%) at moderate risk, and 1 (3%) at high risk. Across most studies, the primary concerns involved inadequate or unclear implementation of participant blinding, intervention provider blinding, allocation concealment, and outcome assessor blinding. These concerns largely reflect the inherent difficulty of blinding behavioural and psychosocial interventions in paediatric settings rather than deficiencies in study conduct per se. Domains relating to participant retention and statistical conclusion validity were well addressed across almost all studies.

**Table 2:**
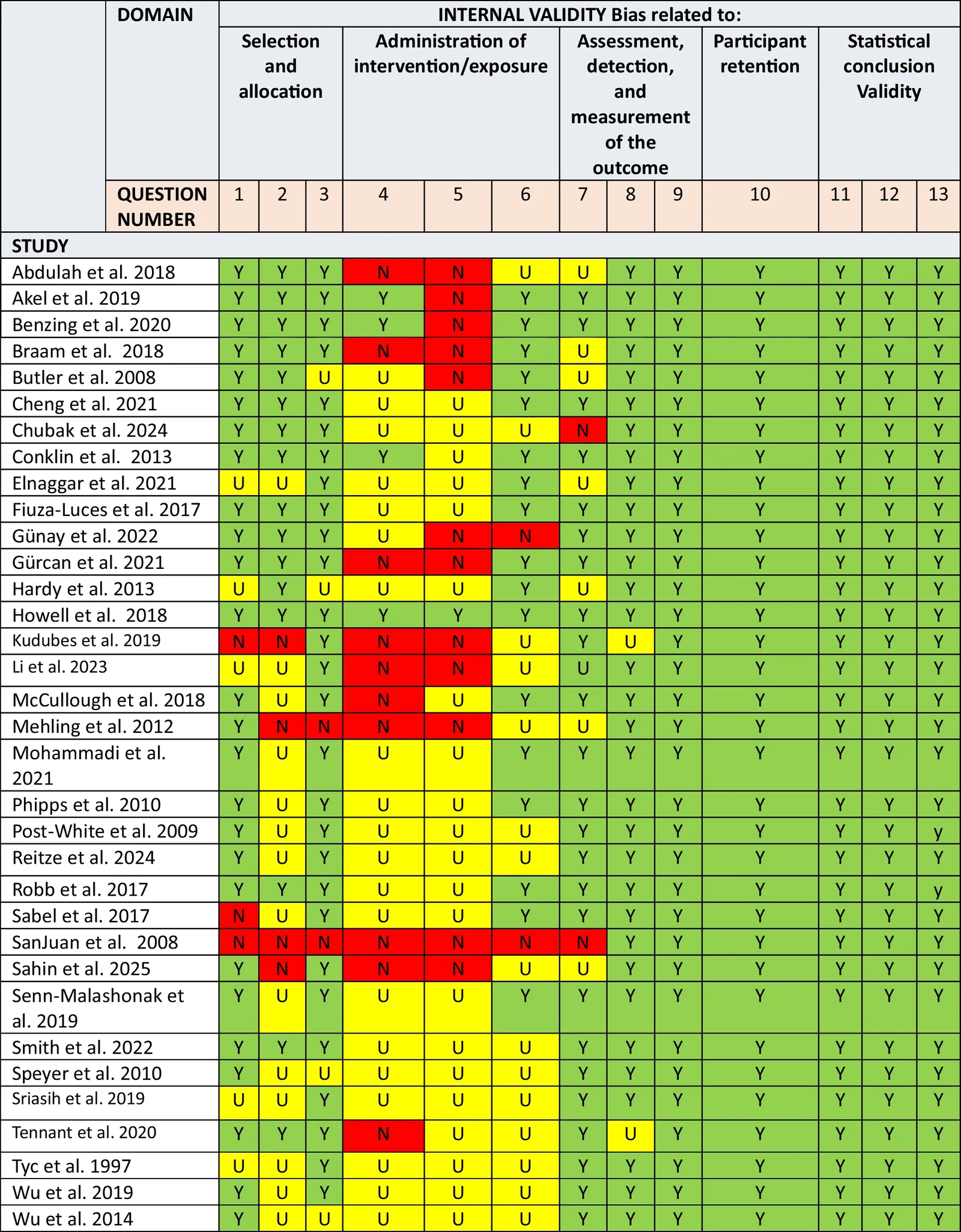
Quality assessment

Question Item: (1) Was true randomization used for assignment of participants to treatment groups? (2) Was allocation to treatment groups concealed? (3) Were treatment groups similar at the baseline? (4) Were participants blind to treatment assignment? (5) Were those delivering treatment blind to treatment assignment? (6) Were outcomes assessors blind to treatment assignment? (7) Were treatment groups treated identically other than the intervention of interest? (8) Was follow up complete and if not, were differences between groups in terms of their follow up adequately described and analyzed? (9) Were participants analyzed in the groups to which they were randomized? (10) Were outcomes measured in the same way for treatment groups? (11) Were outcomes measured in a reliable way? (12) Was appropriate statistical analysis used? (13) Was the trial design appropriate, and any deviations from the standard RCT design (individual randomization, parallel groups) accounted for in the conduct and analysis of the trial?

### Meta-analysis results

There was sufficient data to allow for meta-analyses for neurocognitive outcomes, psychological outcomes, and HRQOL outcomes. Assessment of publication bias was not conducted, as each meta-analysis included fewer than ten studies.

#### Neurocognitive outcomes

**Fig. 2:**
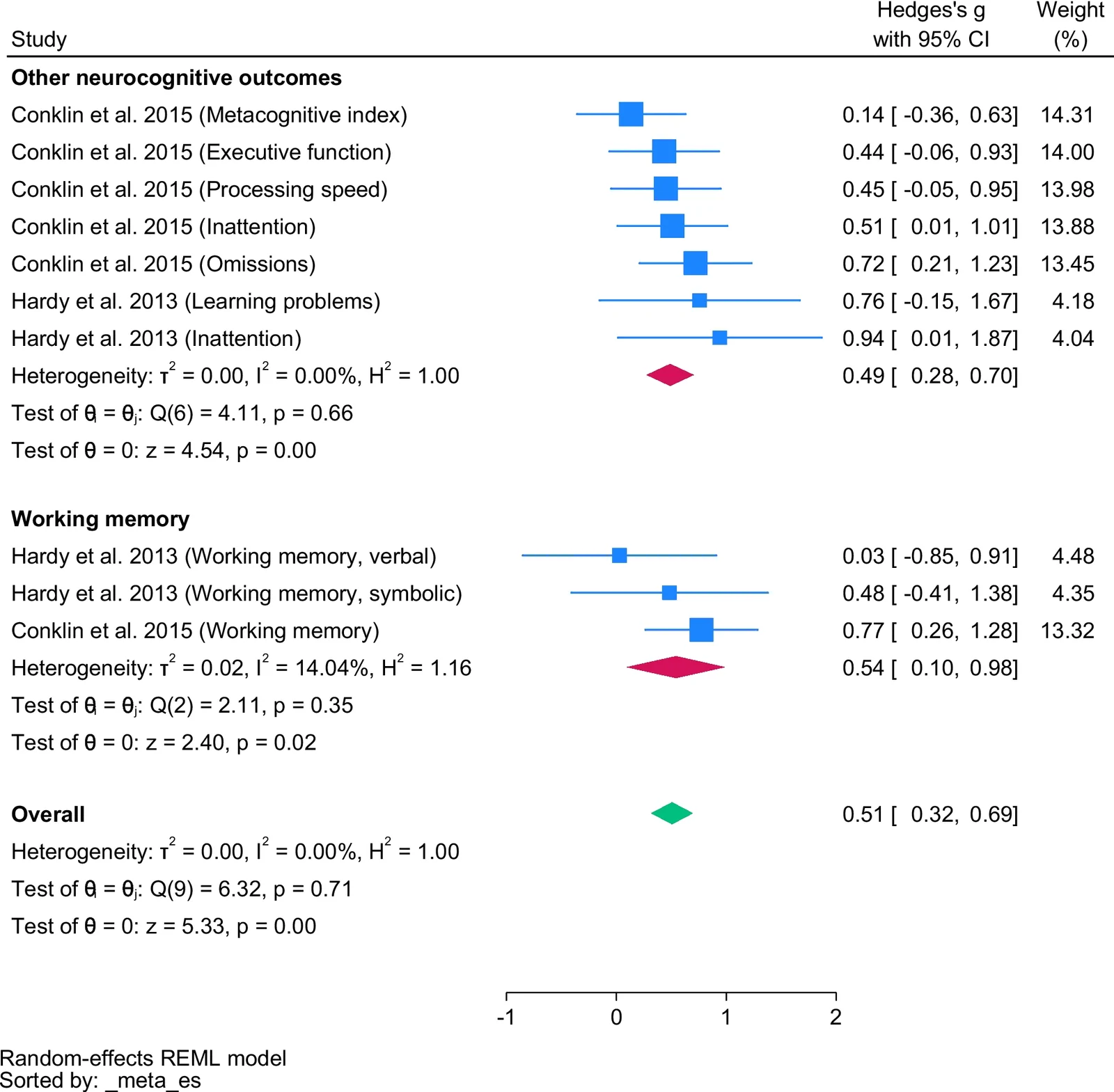
Meta-analysis of neurocognitive outcomes

Meta-analysis results for neurocognitive outcomes are presented in Fig. 2. Overall, studies investigating effects of cognitive interventions demonstrated a significant positive effect on neurocognitive outcomes (SMD: 0.51 [95% CI: 0.32, 0.69]; *I*^2^ = 0%, *p* < 0.01). Ordered by magnitude of effect, significant improvements were observed in working memory (SMD: 0.54 [95% CI: 0.10, 0.98]; *I*^2^ = 14%, *p* = 0.02), followed by a composite of other neurocognitive outcomes, specifically metacognitive skills, executive function, processing speed, inattention, omissions, and learning problems (SMD: 0.49 [95% CI: 0.28, 0.70]; *I*^2^ = 0%, *p* < 0.01).

#### Psychological outcomes

**Fig. 3:**
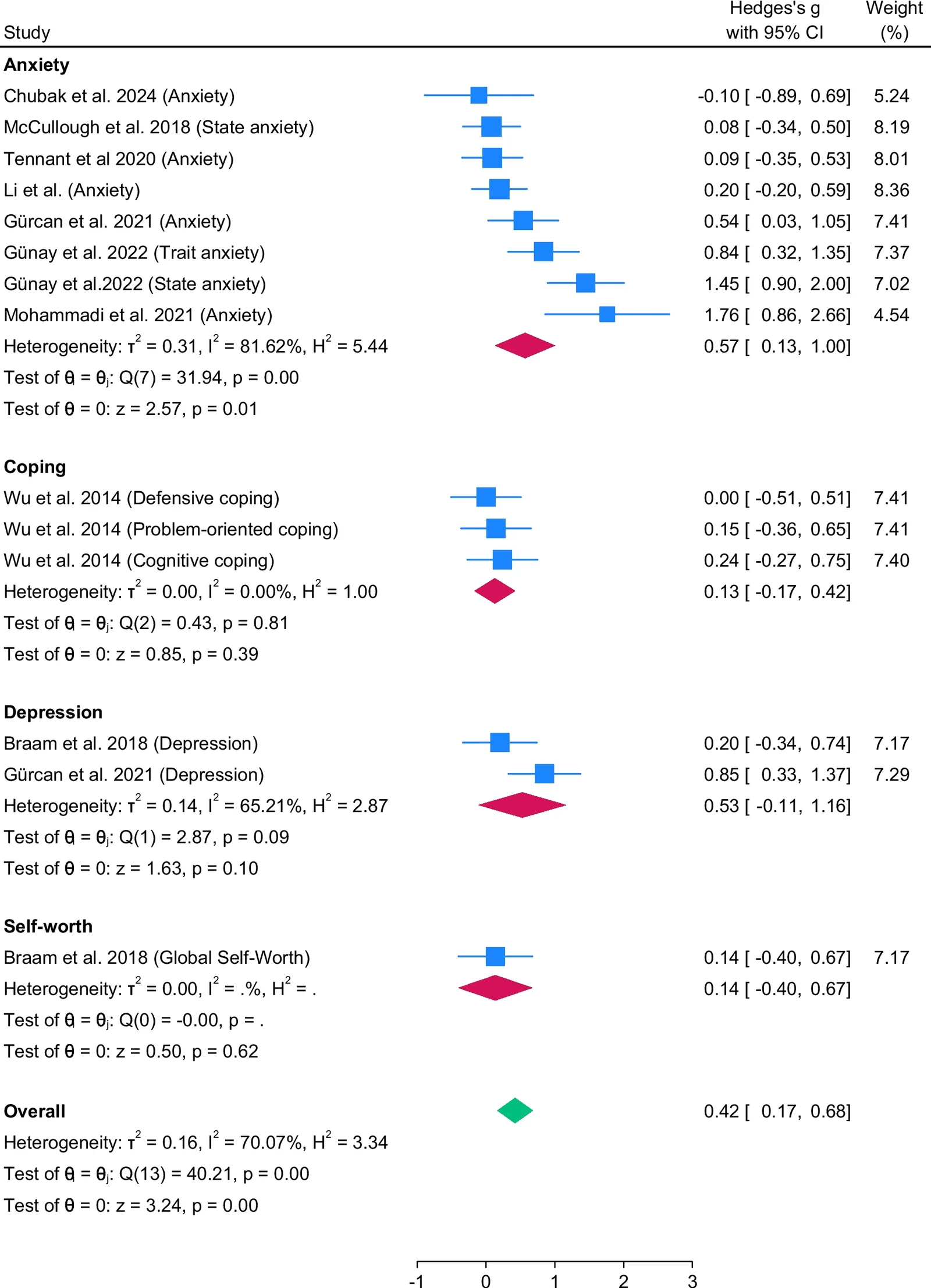
Meta-analysis of psychological outcomes

Figure 3 presents the meta-analysis of psychological outcomes, explored in studies investigating interventions based on play and creative therapies, animal assistance therapy, self-management education, and physical activity. Overall, the interventions were associated with a significant improvement in psychological outcomes (SMD: 0.42 [95% CI: 0.17, 0.68]; *I*^2^ = 70.1%, *p* < 0.01). *I*^2^ values and τ^2^ indicated substantial heterogeneity, suggesting considerable variability in effect sizes across studies. Ordered by magnitude of effect, the largest effect was observed for anxiety, which showed a significant reduction (SMD: 0.57 [95% CI: 0.13, 1.00]; *I*^2^ = 81.6%, *p* < 0.01). No significant effects were observed for depression (SMD: 0.53 [95% CI: –0.11, 1.16]; *I*^2^ = 65.2%, *p* = 0.10), coping (SMD: 0.13 [95% CI: –0.17, 0.42]; *I*^2^ = 0%, *p* = 0.39), or self-worth (SMD: 0.11 [95% CI: –0.40, 0.67]; *I*^2^ = 0%, *p* = 0.62).

#### HRQOL outcomes

**Fig. 4:**
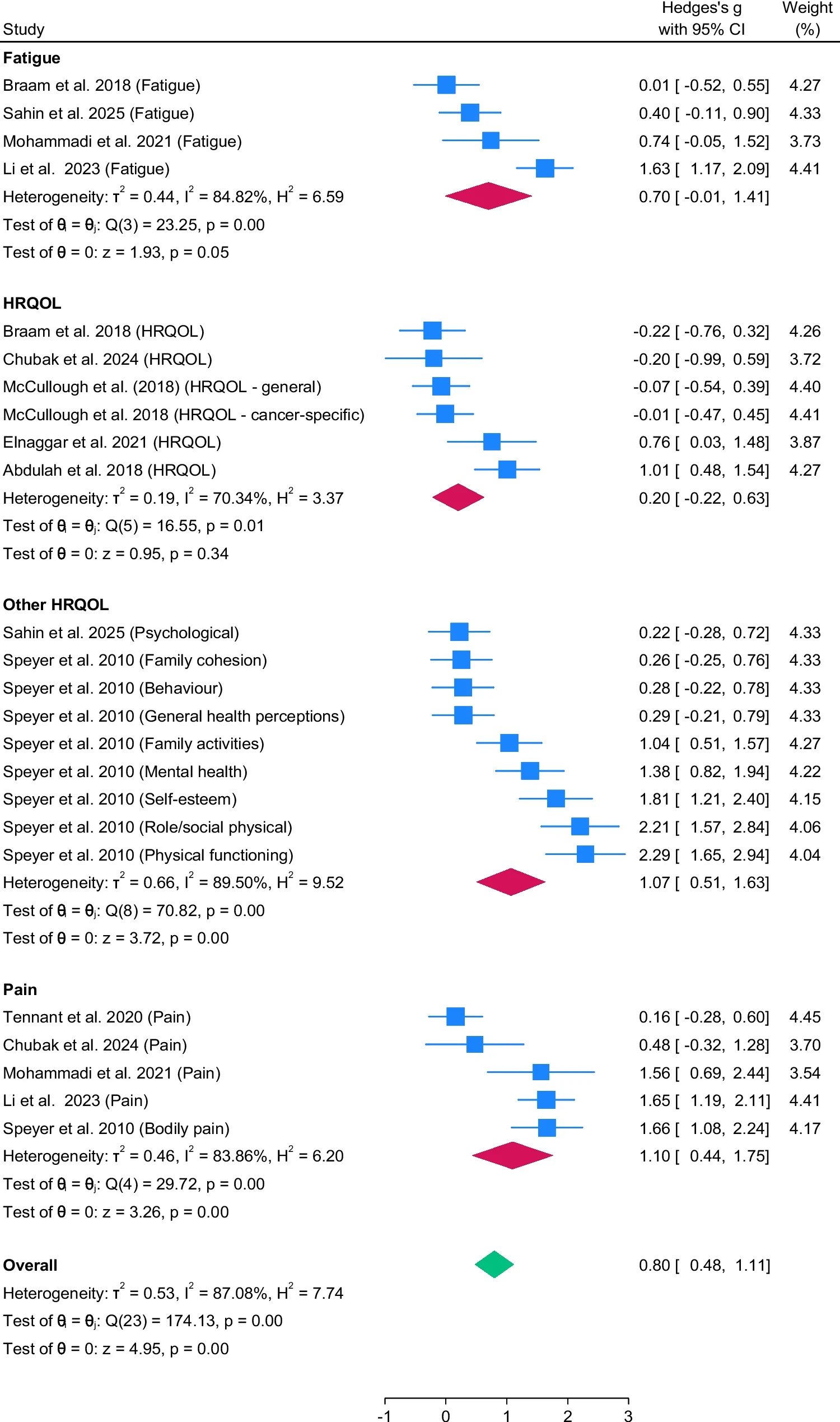
Meta-analysis of HRQOL outcomes

Meta-analysis of HRQOL outcomes is shown in Fig. 4. Interventions included animal assistance therapy, play and creative therapies, and physical activity interventions. Overall, interventions led to a significant improvement in quality-of-life outcomes, as measured by a range of domains (SMD: 0.80 [95% CI: 0.48, 1.11]; *I*^2^ = 87.1%, *p* < 0.01). Ordered by magnitude of effect, significant effects were observed for pain (SMD: 1.10 [95% CI: 0.44, 1.75]; *I*^2^ = 83.9%, *p* < 0.01), followed by other HRQOL-related subdomains (SMD: 1.07 [95% CI: 0.51, 1.63]; *I*^2^ = 89.5%, *p* < 0.01). No significant effects were found for fatigue (SMD: 0.70 [95% CI: −0.01, 1.41]; *I*^2^ = 84.8%, *p* = 0.05) or general HRQOL (SMD: 0.20 [95% CI: −0.22, 0.63]; *I*^2^ = 70.3%, *p* = 0.34). The dispersion of effects was substantial, with *I*^2^ values suggesting significant heterogeneity may be present, and τ^2^ indicating that true effects in comparable populations may vary substantially, reflecting variation in intervention types, outcome measures, and populations.

## Discussion

Children and adolescents (0–19 years) affected by cancer and its treatment are vulnerable to multidimensional challenges spanning neurocognitive function, psychological wellbeing, and broader HRQOL, including treatment-related toxicities, uncertainty about the future, and disruption to family life, schooling, social activity, and sleep during critical periods of development [3]. To our knowledge, this is the largest systematic review and meta-analysis to date of psychosocial interventions across the cancer care continuum for this population [28]. Notably, no trial targeted the pre-treatment (prehabilitation) window, reflecting the urgency with which treatment frequently commences; however, solid-tumour pathways often allow lead time, and Enhanced Recovery After Surgery (ERAS) principles and very early psychosocial input could be initiated through coordinated interprofessional effort as early as is feasible. Because the included interventions varied widely and we pooled at the level of outcome domain rather than intervention, the pooled estimates should be read as evidence of the overall promise of psychosocial intervention as a category rather than precise effects for any single modality [27, 72–76].

The included psychosocial interventions were highly varied in their design, modality, delivery setting and personnel, population, and targeted outcomes. Indicators of heterogeneity suggested that the dispersion of effects across studies was substantial for some outcomes, meaning that the magnitude of benefit varied across interventions and contexts. Although the heterogenous nature of the included interventions precludes definitive conclusions, the results indicated highly promising directions for further research and practice.

### Neurocognitive outcomes

Cognitive interventions produced a consistent, moderate benefit (SMD 0.51; *I*^2^ = 0%), strongest for working memory. Mechanistically, structured, repetition-based cognitive remediation and computerised training plausibly target the working-memory and executive-function processes selectively vulnerable to CNS-impacted therapies, which may explain why benefits concentrated in these domains. Our finding is directionally consistent with He et al. [77] although their broader effects across attention and executive functioning likely reflect wider inclusion criteria; our stricter definition yielded fewer eligible studies, indicating the field is larger than our pooled estimate implies and that the estimate remains provisional. Clinically, cognitive remediation is a credible candidate for integration into survivorship pathways for children treated with CNS-directed therapy, though optimal timing, dose, and delivery format (clinic-based versus home or digital) remain undefined. The broader effects seen in that review may be due to differences in inclusion criteria and/or terminology, resulting in a relatively small number of cognitive intervention studies in our analysis despite evidence that more interventions have been trialled.

### Psychological outcomes

A significant overall benefit (SMD 0.42) was driven primarily by reductions in anxiety; pooled effects on depression, coping, and self-worth were not significant, with substantial heterogeneity (*I*^2^ = 70.1%). A plausible explanation is that several pooled interventions act most directly on acute, state-based arousal around procedures and treatment, to which anxiety is responsive, whereas depression, coping style, and self-worth are more trait-like, evolve over longer horizons, and may require higher-intensity or longer interventions; the non-significant effects are also consistent with insufficient power given few studies per outcome. Compared with earlier syntheses [24, 25], our larger and continuum-wide sample reinforces the anxiety signal while making clearer that effects on mood and self-concept remain unproven. Clinically, brief psychosocial interventions are most promising for state anxiety around treatment and procedures; durable effects on mood and self-concept will require dedicated, theory-driven and adequately powered trials.

### Health-related quality of life

Interventions produced the largest pooled effect (SMD 0.80) but with very high heterogeneity (*I*^2^ = 87.1%); benefits were significant for pain but not fatigue or general HRQOL. The pain finding aligns with evidence that multimodal, non-pharmacological approaches reduce symptom burden. The divergence between a strong domain-specific effect (pain) and a null effect on global HRQOL likely reflects both measurement heterogeneity, meaning generic versus disease-specific instruments capturing different constructs, and the difficulty of shifting a multidimensional construct with single-modality interventions over short durations. Clinically, symptom-targeted approaches (e.g. for procedural pain) are the most promising near-term application, whereas meaningful change in global HRQOL is likely to require sustained, multimodal supportive care models. Wider adoption of paediatric oncology core outcome sets would improve comparability of trial outcomes.

### Implications for research and practice

Our review identified significant evidence gaps in the field of paediatric oncology psychosocial interventions, and clear directions for future opportunities to co-design integrated, interprofessional, multimodal psychosocial models of care as early as possible within the continuum of care. This finding underscores a critical need to explore how early proactive interventions might mitigate treatment-related morbidity and enhance long-term outcomes. While multimodal interventions have shown promise, their efficacy remains underexplored, especially regarding which components are most beneficial, when they should be delivered, and which patient populations are most likely to benefit. Moreover, the underlying mechanisms through which these interventions exert their effects are not well understood, limiting the ability for healthcare professionals and families to tailor approaches to individual needs.

To advance the field, future research should prioritise rigorous evaluation of both early psychosocial interventions (prehabilitation) and rehabilitation (during and post treatment) strategies and could include future explorations around the appropriateness of multimodal designs [78]. Studies should aim to identify optimal intervention timing, dosage, and delivery formats, as well as stratify outcomes by age, diagnosis, and treatment intensity. Importantly, efforts must also be directed toward implementation science to ensure that effective interventions can be feasibly and sustainably translated into routine clinical practice. Building this evidence base will be essential for developing personalised, developmentally appropriate care pathways that support children and adolescents with cancer throughout their treatment journey and beyond.

### Strengths and limitations

This study has several limitations that should be considered when interpreting the findings. The high heterogeneity observed for psychological and HRQOL outcomes is a major limitation. This variability likely reflects differences in intervention modality, intensity, duration, setting, comparator, outcome measure, cancer diagnosis, treatment phase, and developmental age. Although random-effects models account for between-study variability statistically, they do not explain these clinical sources of heterogeneity. Further subgroup or sensitivity analyses were not appropriate because the available studies were too sparse and clinically overlapping to form reliable subgroups. Therefore, pooled estimates should be interpreted as broad evidence of intervention promise, rather than precise estimates for specific intervention types, diagnoses or age groups. Future trials should use clearer intervention classifications, consistent outcome measures, and age- and diagnosis-stratified reporting to enable more robust exploration of heterogeneity.

It is possible that not all relevant studies were captured, particularly non-English language research, which could influence the overall conclusions. We did not separately search grey literature; however our eligibility criteria required the peer-reviewed outcome data necessary for meta-analysis.

Additionally, the broad scope of this review, combined with the heterogeneity of the included studies and the wide range of outcomes assessed, led to substantial variability in intervention types and outcomes. This diversity limited our ability to draw specific conclusions regarding demographic, clinical, or intervention characteristics that might influence outcomes at a micro-level of intervention type classification. The small number of studies per outcome category also limited the ability to explore subgroup effects or publication bias. Developmental age is an important source of clinical heterogeneity in this review. The included studies spanned infancy/early childhood, middle childhood, and adolescence, yet generally did not report age-stratified outcomes or clearly describe developmental tailoring of intervention content. This limits interpretation because psychosocial needs, cognitive capacity, autonomy, family dependence, and peer relationships differ substantially across these stages. Although random-effects models account for statistical heterogeneity, they cannot resolve developmental differences in intervention mechanisms or suitability. Accordingly, the pooled estimates should be interpreted as broad evidence of intervention promise, rather than evidence that effects are consistent across age groups. Future trials should prospectively define developmental age groups, tailor interventions accordingly and report age-stratified outcomes to support more precise synthesis. Publication bias could not be formally assessed because each meta-analysis included fewer than 10 studies, making funnel plot interpretation and tests for small-study effects unreliable. Therefore, publication bias cannot be excluded, and the pooled estimates should be interpreted with this limitation in mind. Finally, many studies included small samples and had some risk of bias, meaning that effect estimates should be interpreted with caution.

## Conclusion

This systematic review and meta-analysis suggest the promise of psychosocial interventions for improving neurocognitive, psychological, and HRQOL outcomes in children and adolescents affected by cancer, while also highlighting substantial evidence gaps, particularly for early targeted approaches in the cancer care continuum. Further rigorous intervention development and testing in trials as well as real-world clinical settings are needed to support more comprehensive and holistic care for this population.

## Supplementary Information

Below is the link to the electronic supplementary material.

ESM 1 (DOCX 21.0 KB)

## Data Availability

No datasets were generated or analysed during the current study.
