# Supplementary material for: Psychosocial interventions for children and adolescents across the cancer care continuum: A systematic review and meta-analysis
Source: Support Care Cancer. 2026 Sep 21;34(10):995. doi: 10.1007/s00520-026-11176-x (PMC13593794; doi:10.1007/s00520-026-11176-x)
Supplement: Supplementary file 1 [file 520_2026_11176_MOESM1_ESM.docx]

**Supplement 1 – Database searches**

Databases were searched on 17 November 2025 to identify relevant studies. Full record of searches below.

**APA PsycINFO (via EBSCOhost)**

| Search | Search concept | Search terms | Results |
| --- | --- | --- | --- |
| #1 | Prehabilitation / Rehabilitation | prehab* OR “pre-operative conditioning” OR “preoperative conditioning” OR “pre-operative exercise*” OR “preoperative exercise*” OR “pre-operative rehabilitation” OR “preoperative rehabilitation” OR “peri-operative exercise*” OR “perioperative exercise*” OR “peri-operative rehabilitation” OR “perioperative rehabilitation” OR ((pre-op* OR preop* OR peri-op* OR periop* OR rehab*) N50 (diet* OR exercise OR food OR “group therapy” OR intervention OR mental OR nutrition* OR online OR physical OR psycho* OR “self-help” OR “skills training” OR “support group” OR virtual OR web-based OR wellbeing)) | 57,380 |
| #2 | Children and adolescents | adolescent* OR child* OR paediatric OR pediatric OR teen* OR young OR youth | 1,597,580 |
| #3 | Cancer | cancer* OR malignan* OR neoplasm* OR oncolog* | 125,451 |
| #4 | Randomised Controlled Trials | randomi?ed OR randomly OR RCT OR trial | 353,613 |
| #5 |  | #1 AND #2 AND #3 AND #4 | 70 |

**CENTRAL – The Cochrane Central Register of Controlled Trials**

| Search | Search concept | Search terms | Results |
| --- | --- | --- | --- |
| #1 |  | "Preoperative Exercise" [MeSH] | 180 |
| #2 |  | "Rehabilitation" [MeSH] | 58,263 |
| #3 |  | prehab* OR (pre-operative NEXT conditioning) OR (preoperative NEXT conditioning) OR (pre-operative NEXT exercise*) OR (preoperative NEXT exercise*) OR (pre-operative NEXT rehabilitation) OR (preoperative NEXT rehabilitation) OR (peri-operative NEXT exercise*) OR (perioperative NEXT exercise*) OR (peri-operative NEXT rehabilitation) OR (perioperative NEXT rehabilitation) OR ((pre-op* OR preop* OR peri-op OR periop* OR rehab*) NEAR/50 (diet* OR exercise OR food OR (group NEXT therapy) OR intervention OR mental OR nutrition* OR online OR physical OR psycho* OR self-help OR (skills NEXT training) OR (support NEXT group) OR virtual OR web-based OR wellbeing)) | 80,049 |
| #4 | Prehabilitation / Rehabilitation | #1 OR #2 OR #3 | 15120,772 |
| #5 |  | "Child” [MeSH] | 83,695 |
| #6 |  | "Adolescent" [MeSH] | 138,990 |
| #7 |  | adolescent* OR child* OR paediatric OR pediatric OR teen* OR young OR youth | 463,879 |
| #8 | Children and adolescents | #5 OR #6 OR #7 | 463,879 |
| #9 |  | "Neoplasms” [MeSH] | 130,416 |
| #10 |  | cancer* OR malignan* OR neoplasm* OR oncolog* | 295,577 |
| #11 | Cancer | #9 OR #10 | 307,215 |
| #12 |  | #4 AND #8 AND #11 | 1,445 |
|  |  | Limiter: Trials  PubMed – 795  Embase - 461  ICTRP - 82  CT.gov - 63  CINAHL – 7  TOTAL – 1,038 | 1,038 |

**ClinicalTrials.gov**

| Search | Search concept | Search terms | Results |
| --- | --- | --- | --- |
| #1 |  | Condition: Cancer  Other terms: (prehab* OR rehab*) AND children  Study status: Completed | 82 |

**CINAHL (via EBSCOhost)**

| Search | Search concept | Search terms | Results |
| --- | --- | --- | --- |
| #1 | Prehabilitation / Rehabilitation | (MH "Prehabilitation") OR (MH "Rehabilitation+") OR prehab* OR “pre-operative conditioning” OR “preoperative conditioning” OR “pre-operative exercise*” OR “preoperative exercise*” OR “pre-operative rehabilitation” OR “preoperative rehabilitation” OR “peri-operative exercise*” OR “perioperative exercise*” OR “peri-operative rehabilitation” OR “perioperative rehabilitation” OR ((pre-op* OR preop* OR peri-op* OR periop* OR rehab*) N50 (diet* OR exercise OR food OR “group therapy” OR intervention OR mental OR nutrition* OR online OR physical OR psycho* OR “self-help” OR “skills training” OR “support group” OR virtual OR web-based OR wellbeing)) | 411,241 |
| #2 | Children and adolescents | (MH "Child+") OR (MH "Adolescence+") OR adolescent* OR child* OR paediatric OR pediatric OR teen* OR young OR youth | 1,775,556 |
| #3 | Cancer | (MH "Neoplasms+") OR cancer* OR malignan* OR neoplasm* OR oncolog* | 951,168 |
| #4 | Randomised Controlled Trials | (PT "Randomized Controlled Trial") OR randomi?ed OR randomly OR RCT OR trial | 742,328 |
| #5 |  | #1 AND #2 AND #3 AND #4 | 550 |

**MEDLINE – (via EBSCOhost)**

| Search | Search concept | Search terms | Results |
| --- | --- | --- | --- |
| #1 | Prehabilitation / Rehabilitation | (MH "Preoperative Exercise") OR (MH "Rehabilitation+") OR prehab* OR “pre-operative conditioning” OR “preoperative conditioning” OR “pre-operative exercise*” OR “preoperative exercise*” OR “pre-operative rehabilitation” OR “preoperative rehabilitation” OR “peri-operative exercise*” OR “perioperative exercise*” OR “peri-operative rehabilitation” OR “perioperative rehabilitation” OR ((pre-op* OR preop* OR peri-op* OR periop* OR rehab*) N50 (diet* OR exercise OR food OR “group therapy” OR intervention OR mental OR nutrition* OR online OR physical OR psycho* OR “self-help” OR “skills training” OR “support group” OR virtual OR web-based OR wellbeing)) | 584,174 |
| #2 | Children and adolescents | (MH "Child+") OR (MH "Adolescent") OR adolescent* OR child* OR paediatric OR pediatric OR teen* OR young OR youth | 6,202,087 |
| #3 | Cancer | (MH "Neoplasms+") OR cancer* OR malignan* OR neoplasm* OR oncolog* | 5,874,025 |
| #4 | Randomised Controlled Trials | (PT "Randomized Controlled Trial") OR randomi?ed OR randomly OR RCT OR trial | 2,438,440 |
| #5 |  | #1 AND #2 AND #3 #4 | 1,452 |

**Scopus**

| Search | Search concept | Search terms | Results |
| --- | --- | --- | --- |
| #1 | Prehabilitation / Rehabilitation | prehab* OR “pre-operative conditioning” OR “preoperative conditioning” OR “pre-operative exercise*” OR “preoperative exercise*” OR “pre-operative rehabilitation” OR “preoperative rehabilitation” OR “peri-operative exercise*” OR “perioperative exercise*” OR “peri-operative rehabilitation” OR “perioperative rehabilitation” OR ((pre-op* OR preop* OR peri-op* OR periop* OR rehab*) W/50 (diet* OR exercise OR food OR “group therapy” OR intervention OR mental OR nutrition* OR online OR physical OR psycho* OR “self-help” OR “skills training” OR “support group” OR virtual OR web-based OR wellbeing)) | 219,609 |
| #2 | Children and adolescents | adolescent* OR child* OR paediatric OR pediatric OR teen* OR young OR youth | 7,231,672 |
| #3 | Cancer | cancer* OR malignan* OR neoplasm* OR oncolog* | 5,829,906 |
| #4 | Randomised Controlled Trials | randomi?ed OR randomly OR RCT OR trial | 4,090,327 |
| #5 |  | #1 AND #2 AND #3 AND #4 | 546 |
